# A Prospective Analysis of Lesion-Symptom Relationships in Acute Vestibular and Ocular Motor Stroke

**DOI:** 10.3389/fneur.2020.00822

**Published:** 2020-08-06

**Authors:** Andreas Zwergal, Ken Möhwald, Elvira Salazar López, Hristo Hadzhikolev, Thomas Brandt, Klaus Jahn, Marianne Dieterich

**Affiliations:** ^1^Department of Neurology, University Hospital, LMU Munich, Munich, Germany; ^2^German Center for Vertigo and Balance Disorders, DSGZ, LMU Munich, Munich, Germany; ^3^Department of Human Movement Science, Technical University of Munich, Munich, Germany; ^4^Clinical Neurosciences, LMU Munich, Munich, Germany; ^5^Department of Neurology, Schön Klinik Bad Aibling, Bad Aibling, Germany; ^6^Munich Cluster of Systems Neurology, SyNergy, Munich, Germany

**Keywords:** vertigo, dizziness, double vision, acute vestibular syndrome, stroke

## Abstract

**Background:** Diagnosing stroke as a cause of acute vertigo, dizziness, or double vision remains a challenge, because symptom characteristics can be variable. The purpose of this study was to prospectively investigate lesion-symptom relationships in patients with acute vestibular or ocular motor stroke.

**Methods:** Three hundred and fifty one patients with acute and isolated vestibular or ocular motor symptoms of unclear etiology were enrolled in the EMVERT lesion trial. Symptom quality was assessed by the chief complaint (vertigo, dizziness, double vision), symptom intensity by the visual analog scale, functional impairment by EQ-5D-5L, and symptom duration by daily rating. Acute vestibular and ocular motor signs were registered by videooculography. A standardized MRI (DWI-/FLAIR-/T2-/T2^*^-/3D-T1-weighted sequences) was recorded within 7 days of symptom onset. MRIs with DWI lesions were further processed for voxel-based lesion-symptom mapping (VLSM).

**Results:** In 47 patients, MRI depicted an acute unilateral stroke (13.4%). The chief complaints were dizziness (42.5%), vertigo (40.4%) and double vision (17.0%). Lesions in patients with vertigo or dizziness showed a large overlap in the cerebellar hemisphere. VLSM indicated that strokes in the medial cerebellar layers 7b, 8, 9 were associated with vertigo, strokes in the lateral cerebellar layer 8, crus 1, 2 with dizziness, and pontomesencephalic strokes with double vision. Symptom intensity and duration varied largely between patients. Higher symptom intensity and longer duration were associated with medial cerebellar lesions. Hemispheric lesions of the cortex were rare and presented with milder symptoms of shorter duration.

**Conclusions:** Prospective evaluation of patients with acute vestibular or ocular motor stroke revealed that symptom quality, intensity and duration were not suited to differentiating peripheral from central etiologies. Lesions in the lateral cerebellum, thalamus, or cortex presented with unspecific, mild and transient symptoms prone to being misdiagnosed.

## Introduction

Vertigo, dizziness or double vision may be symptoms of an acute cerebral ischemia or hemorrhage (1). Overall, 4–10% of patients in the emergency department (ED) presenting with vertigo and balance disorders suffer from stroke (2). Sixteen percentage of diplopia-related ED visits result from stroke or TIA (3). Patients with vestibular or ocular motor stroke often have no additional focal neurological deficits and therefore are at greater risk of being misdiagnosed (4, 5).

Cerebral lesions presenting with vertigo, dizziness, or double vision mostly involve vestibular and ocular motor circuits in the brainstem and cerebellum, whereas thalamo-cortical networks are affected only occasionally (6, 7). The reason for this lesion distribution can be found in the functional anatomy of the bilaterally organized central vestibular system, which converts direction-specific signals of each labyrinth into more global position-in-space signals along the ascending vestibular projections (8). Consequently, the vestibular syndromes of the lower brainstem present with severe vertigo and ipsilesional falling tendency, while lesions of the parieto-insular vestibular cortex may cause “higher vestibular symptoms” such as altered spatial perception or neglect (9). However, previous knowledge about the topography and symptoms of pure vestibular or ocular motor strokes is mostly based on retrospective analyses, which lack detail in the description of the quality, intensity, and time course of clinical symptoms.

Therefore, in the prospective EMVERT (EMergency VERTigo) lesion trial, symptoms and lesion topography were characterized in consecutive patients, who presented to the ED of a tertiary referral center with acute vertigo, dizziness or double vision due to stroke (10). This approach focuses on the clinical triage practice in the ED, which initially is based on the description of symptoms by the patient rather than on vestibular and ocular motor signs. The major question was whether symptom characteristics could be sufficient to differentiate peripheral from central disorders. A further aim was the evaluation of the distribution of lesion sites in pure vestibular and ocular motor stroke in relation to the quality, intensity, and time course of the accompanying chief complaint.

## Methods

### Patient Characteristics

**Figure 1:**
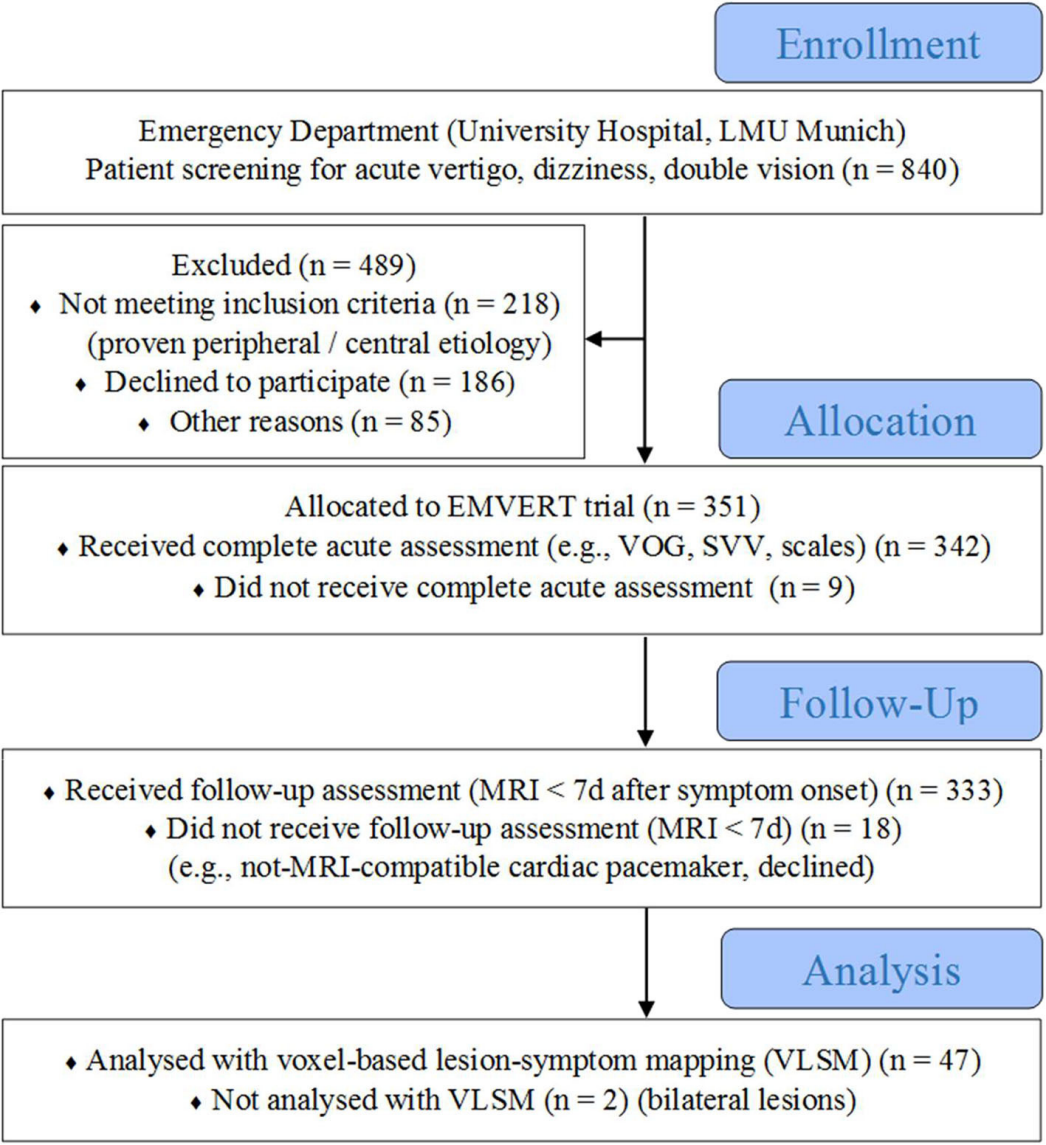
Flow diagram of the EMVERT lesion trial. Eight hundred and forty consecutive patients with an acute presentation of vertigo, dizziness, or double vision were prospectively screened for inclusion in the EMVERT lesion trial at the Emergency Department (ED) of the University Hospital, Ludwig-Maximilians-University, Munich. Four hundred and eighty nine patients were excluded, because of the following reasons: definite peripheral vestibular or ocular motor disorder (like definite BPPV, recurrent attacks of definite Menière's disease, definite peripheral N III, N IV, N VI palsy) (*n* = 203); stroke with accompanying non-vestibular symptoms (like hemiparesis, hemihypesthesia, hemiataxia) (*n* = 15); decline to participate (*n* = 186); incapability to be included for other reasons (e.g., communications problem, psychiatric co-morbidity, cognitive deficits, critical illness, symptoms < 10 min) (*n* = 85). Three hundred and fifty one patients (60.1 ± 16.7 years, 46.6% female) with isolated vertigo, dizziness or double vision of unclear etiology were included. Three hundred and forty two patients received complete acute assessment in the ED, 333 patients a follow-up MRI within 7 days after symptom onset. Forty-seven of 49 patients with a stroke on MRI were included in the voxel-based lesion-symptom mapping analysis (2 excluded because of bilateral lesions). MRI, magnetic resonance imaging; SVV, subjective visual vertical; VLSM, voxel-based lesion-symptom mapping; VOG, videooculography.

Eight hundred and forty consecutive patients with an acute presentation of vertigo, dizziness or double vision were prospectively screened for inclusion in the EMVERT lesion trial at the ED of the University Hospital, Ludwig-Maximilians-University, Munich. Four hundred and eighty-nine patients were excluded, because of the following reasons: definite peripheral vestibular or ocular motor disorders (like nystagmus typical for BPPV during repositioning maneuvers, recurrent attacks of definite Menière's disease, definite peripheral N III, N IV, N VI palsy without central ocular motor signs, vertigo/dizziness, or SVV deviation on the non-paretic eye) (*n* = 203); strokes with accompanying non-vestibular symptoms (like hemiparesis, hemihypesthesia, hemiataxia) (*n* = 15); decline to participate (*n* = 186); incapability to be included for other reasons (e.g., communications problem, psychiatric co-morbidity, cognitive deficits, critical illness, symptoms <10 min) (*n* = 85). Three hundred and fifty-one patients (60.1 ± 16.7 years, 46.6% female) with isolated vertigo, dizziness or double vision of unclear etiology were included (Figure 1). Two hundred and sixty patients had persistent symptoms at the time of inclusion. Fifty eight percentage of symptomatic patients had spontaneous nystagmus (SPN) at acute examination.

### Protocol Approval and Patient Consent

The study was approved by the Ethics Committee of the University of Munich on 02/23/2015 (57-15). The study was conducted according to the Guideline for Good Clinical Practice, the Federal Data Protecting Act and the Helsinki Declaration of the World Medical Association (revision of Fortaleza, Brazil, October 2013). All subjects gave their informed, written consent to participate in the study.

### Trial Flow

Adult patients with an acute onset of vertigo, dizziness, or double vision within the last 24 h and a duration of at least 10 min were screened prospectively for inclusion in this single center trial (10). A structured medical history and standardized clinical examination with an emphasis on vestibular and ocular motor function tests was performed in the ED. Patients with a clinically proven peripheral etiology (e.g., typical signs of BPPV, recurrent attacks of definite Menière's disease, peripheral N III, N IV, N VI palsy), and central etiology (e.g., signs of acute hemiparesis, hemihypesthesia, hemiataxia) were excluded. The remaining patients with unclear etiology of isolated vertigo, dizziness or double vision were defined as the subpopulation of interest for the EMVERT trial. Patients, who consented to participate, were included and received a comprehensive assessment of vestibular, ocular motor and postural signs by videooculography (VOG, EyeSeeCam®), mobile posturography, measurement of SVV, as well as scores and scales in the ED. A standardized magnetic resonance imaging (MRI) protocol was applied within 7 days after symptom onset to identify acute stroke (time to MRI: 2 ± 2.8 days, 93% of cases > 1 day) (Figure 1).

### Scoring and Scaling of Chief Complaint, Symptom Duration, and Functional Impairment

At admission, the patients were asked to categorize their chief complaint as either vertigo (sensation of apparent self-motion), dizziness (unspecific sensation without self-motion), or double vision. If mixed phenotypes (e.g., vertigo/double vision) were reported, the patient had to choose the predominant one. Accompanying vegetative symptoms like nausea or vomiting were documented. The maximum intensity of the chief complaint was measured using a visual analog scale (VAS, range 0–10). Decline of symptoms intensity was estimated by repeated testing of VAS for the chief complaint. Duration of vestibular or ocular motor symptoms was categorized in <1 days, 1–4 days, and >4 days based on daily reports of the patients. The Dizziness Handicap Inventory (DHI) and European Quality of Life scale-5 dimensions-5 levels (EQ-5D-5L) were performed as additional scores for graduation of symptom severity, quality of life (QoL) and functioning at admission (11). The Modified Ranking Scale (mRS) was documented at the time of discharge from the hospital.

### Assessment of Vestibular and Ocular Motor Signs

The following vestibular and ocular motor signs were documented by VOG in the ED: nystagmus in straight ahead position (slow-phase velocity with/without fixation), gaze holding (lateral/vertical gaze positions), smooth pursuit (horizontal/vertical direction), saccades (horizontal/vertical direction), horizontal vestibulo-ocular reflex (VOR) (gain threshold: 0.7, presence of compensatory saccades), horizontal VOR-suppression, skew deviation and ocular motility deficits (cover test in lateral, vertical and straight ahead gaze position). The main criterion of skew deviation (in contrast to vertical misalignment due to N III or N IV palsy) was that the amount of vertical deviation from both eyes was the same in different eye positions on alternating cover test. VOG recording was done at the non-paretic eye, if monocular motility was restricted. Binocular subjective visual vertical (SVV) was measured in general, using the bucket test. SVV was determined via the non-affected eye in case of monocular paretic eye movements. Ten repetitions were performed (5 clockwise, 5 counterclockwise) and the mean SVV deviation was calculated (normal range: 0 ± 2.5°) (12).

### MRI Protocol

The standardized protocol included whole brain and brainstem fine slice (3 mm) DWI, FLAIR-, T2-, T2^*^-weighted images, 3D-T1-weighted sequences (FSPGR 1 mm) and time-of-flight angiography. All images were evaluated for the presence of ischemic stroke or bleeding by two specialized neuro-radiologists.

### Voxel-Based Lesion-Symptom Mapping

Lesions were directly manually delineated on DWI sequences (MRI < 3 days post stroke) or T2-weighted sequences (MRI > 3 days post stroke) by an experienced imaging scientist, blinded for the clinical information, on a slice-by-slice basis using MRIcron (13). DWI or T2-images were co-registered with 3D-T1 images to enrich the normalization process. Normalization quality of lesion maps was visually checked by a second operator. Right-sided lesions were flipped to the left for the purpose of analysis. Patients presenting with bilateral lesions (*n* = 2) were discarded for analysis. Patients with simultaneous lesions in the medial and lateral cerebellum, in the medulla and cerebellum and in multiple unilateral locations were included in the analysis. None of the patient had critical ischemic edema. Images were normalized to Montreal Neurological Institute space (MNI) by Statistical Parametric Mapping Software (SPM 8) employing an established template. For descriptive analysis, the lesion site was assigned to the respective vascular territory/territories [posterior inferior cerebellar artery (PICA), anterior inferior cerebellar artery (AICA), superior cerebellar artery (SCA), brainstem perforators, middle cerebral artery (MCA), posterior cerebral artery (PCA)] and the affected anatomical structure(s) (cerebellar midline: nodulus, uvula, pyramis, tonsil, lingula, central lobule; cerebellar hemispheres: flocculus, biventer, inferior/superior semilunar, posterior/anterior quadrangulate lobule; brainstem: medulla, pons, midbrain; thalamus: dorsolateral, anteromedial; cortex: parieto-insular cortex, occipital cortex).

Voxel-based lesion-symptom mapping (VLSM) was performed using the statistical package Non-Parametric Mapping (NPM) implemented in MRIcron. For lesion analysis a custom-made mask was applied (Supplement 1), which included all relevant hubs of the cerebral vestibular network (e.g., brainstem, cerebellum, thalamus and insula). *T-*test (numerical variables) or *Liebermeister*-test (dichotomous variables) corrected for multiple comparison with false discovery rate (FDR) were calculated to assess whether behavioral scores differed significantly between the patients' pattern for lesioned and non-lesioned voxels (14). Since NPM toolbox interprets that a lower value in the behavioral scoring refers to a poorer performance, the different behavioral scores were computed reversed when necessary for statistical purposes. Only voxels affected in 15% of the sample were computed in each analysis to avoid inflated *z*-scores. Areas with significant differences in VLSM were labeled using the Automated Anatomical Labeling template (AAL-Atlas) (15).

### Statistics

ANOVA with *post-hoc* testing was used to compare the scoring and scaling data (e.g., lesion volume, VAS) between subgroups (e.g., stroke/non-stroke, left-/right-sided lesions, vertigo/dizziness) using SPSS®24 (IBM). Pearson's correction coefficient was calculated for the correlation of lesion volume and VAS in the total group and subgroups (vertigo, dizziness, double vision).

### Data Availability

Data reported in this article will be shared with any appropriately qualified investigator on request after pseudonymization.

## Results

### Patient Characteristics

MRI indicated acute unilateral stroke in 47 patients (13.4% of enrolled patients, 5.6% of screened patients, 29 men). The mean age of stroke patients was 64.7 ± 13.0 years. The most frequent chief complaint in stroke patients was dizziness (42.5%), followed by vertigo (40.4%) and double vision (17.0%). Fifty percentage of patients with the chief complaint double vision reported accompanying vertigo or dizziness. 40.4% of stroke patients had nausea or vomiting, none had hiccups. Age did not differ significantly between the subgroups dizziness (66.7 ± 14.2 years), vertigo (63.5 ± 13.2 years), and double vision (61.9 ± 8.0 years). Forty four patients with vestibular or ocular motor stroke were symptomatic at the time of acute VOG assessment. In these patients spontaneous nystagmus (SPN) was detected in 45%. In total 74% of patients with vertigo and 30% of patients with dizziness had SPN (Table 1). In these cases, HINTS had a central pattern in 93% vs. 83% of patients (vertigo vs. dizziness). The head impulse test (HIT) was normal in 95, 85, and 63% of cases (vertigo, dizziness and double vision). Skew deviation appeared in 26% of patients with vertigo, 20% of patients with dizziness and 25% of patients with double vision. SVV was pathological in 68, 65, and 88% of patients with vertigo, dizziness, and double vision. The etiologies of double vision were internuclear ophthalmoplegia (37%), skew deviation (25%) and N III, N IV, N VI nuclear/fascicular palsy (13%, each) (Table 1).

**Table 1:** Vestibular and ocular motor signs in patients with stroke.

	**Vertigo (%)**	**Dizziness (%)**		**Double Vision (%)**
SPN	74	30	Oculomotor palsy	13
HINTS central^*^	68	25	Trochlear palsy	13
HIT normal	95	85	Abducens palsy	13
Skew deviation	26	20	INO	37
SVV	68	65	Skew deviation	25

*An acute vestibular syndrome with SPN was more frequent in patients with vertigo compared to dizziness. HINTS had a high diagnostic sensitivity, if SPN was present, irrespective of the chief complaint. Data are shown as % of all patients with the respective chief complaint. HINTS, head impulse, nystagmus, test of skew; HIT, head impulse test; INO, internuclear ophthalmoplegia; SPN, spontaneous nystagmus; SVV, subjective visual vertical*.

* *HINTS is supposed to be applied only in patients with SPN. The reported HINTS sensitivity in the table is irrespective of the presence of SPN*.

### Lesion Topography and Chief Complaint

**Figure 2:**
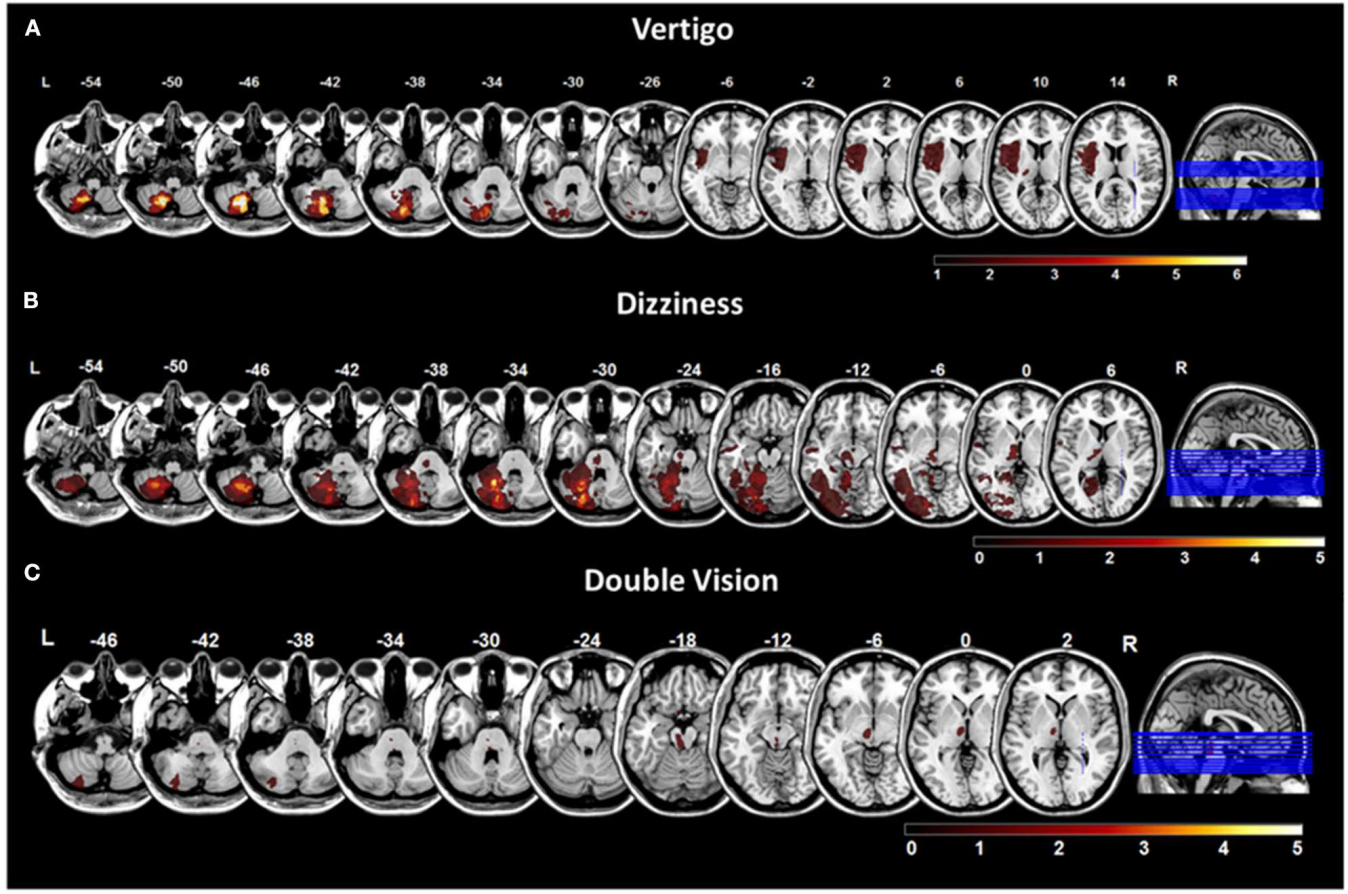
Overlap lesion plots for chief complaints. Overlap lesion plots for the chief complaints vertigo **(A)**, dizziness **(B)**, and double vision **(C)**. The number of overlapping lesions is illustrated by the color bar from dark red (*n* = 0) to bright yellow (maximum number). Coordinates are given in MNI space. MNI, Montreal Neurological Institute; L, left; R, right.

In the total group, the most common lesion sites were in the cerebellum (PICA > SCA territory), followed by the brainstem (pontomedullary > mesencephalic tegmentum), thalamus and cortex. Lesion volume did not differ in patients with right-sided (mean: 8.0 cc, range 0.01–33.6 cc) and left-sided lesions (mean: 8.6 cc, range 0.01–102.2 cc) (*p* = 0.89). Patients with vertigo most frequently had lesions in the medial PICA territory (biventer lobule 58%, inferior semilunar lobule 37%, nodulus 37%, uvula 32%, tonsil 32%) and the pontomedullary brainstem (medulla 16%, pons 21%) (Figure 2A, Supplement 2). In patients with dizziness the lesions were found mostly in the lateral PICA territory (biventer lobule 25%, superior semilunar lobule 25%), SCA territory (posterior/anterior quadrangulate lobule 15%, each), the pontomesencephalic brainstem tegmentum (midbrain 25%, pons 20%) and the thalamus (dorsolateral/anteromedial 5%, each) (Figure 2B, Supplement 2). Lesions of patients with vertigo and dizziness showed a considerable overlap in the PICA territory (biventer, inferior semilunar lobule). Patients with double vision had pontomesencephalic and mesodiencephalic lesions (Figure 2C). Lesion volume was different in patients with vertigo (13.2 ± 24.3 cc), dizziness (7.4 ± 7.7 cc) and double vision (0.5 ± 0.6 cc) (*p* = 0.04). Patients with strokes in the medial cerebellum (PICA territory) had nausea or vomiting in 91%, in the lateral cerebellum, pontomesencephalic brainstem and thalamus in only 17%, respectively. Symptomatic stroke patients without SPN (*n* = 24) had lesions in the lateral PICA territory (30%), medial PICA territory (8%), SCA territory (8%), pontomesencephalic brainstem (38%), thalamus (8%), and insular cortex (8%).

**Figure 3:**
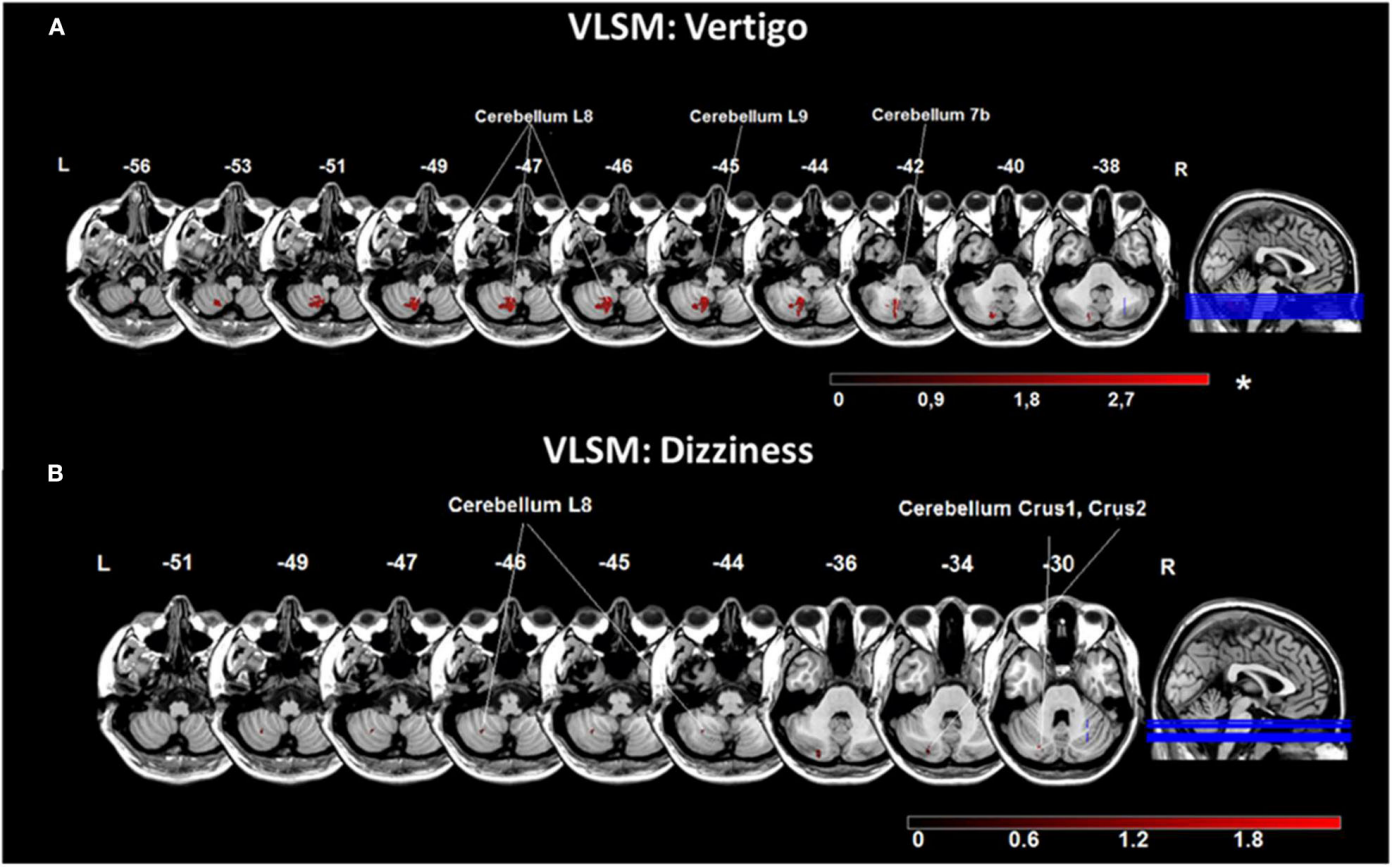
Voxel-based lesion-symptom mapping (VLSM) analysis for the chief complaints vertigo and dizziness. **(A)** VLSM for the presence of the chief complaint vertigo showed an association with areas in the medial cerebellar hemisphere (cerebellar layers 7b, 8, 9). **(B)** VLSM analysis for the chief complaint dizziness indicated related voxels more laterally in the cerebellar hemisphere (cerebellar layer 8, Crus 1, 2). Presented are voxels that exceed a *p* < 0.05 (*FDR-corrected for vertigo; uncorrected for dizziness). The color bar from dark to light red indicates the z-scores. Coordinates are given in MNI space. MNI, Montreal Neurological Institute; L, left; R, right.

VLSM in vestibular networks revealed that lesions in the medial cerebellar layers 7b, 8, 9 were significantly associated with the vertigo [*Liebermeister-*test, *p* = 0.05 (FDR-corrected), Z = 1.66] (Figure 3A). For the chief complaint dizziness, a lesion core area was found in the lateral cerebellar layer 8 and Crus 1, 2 using VLSM [*Liebermeister-*test, *p* < 0.05 (uncorrected), Z = 0.65] (Figure 3B). VLSM analysis conducted in patients with double vision did not reveal a significant association to a certain brain area (data not shown).

### Lesion Topography and Symptom Intensity

**Figure 4:**
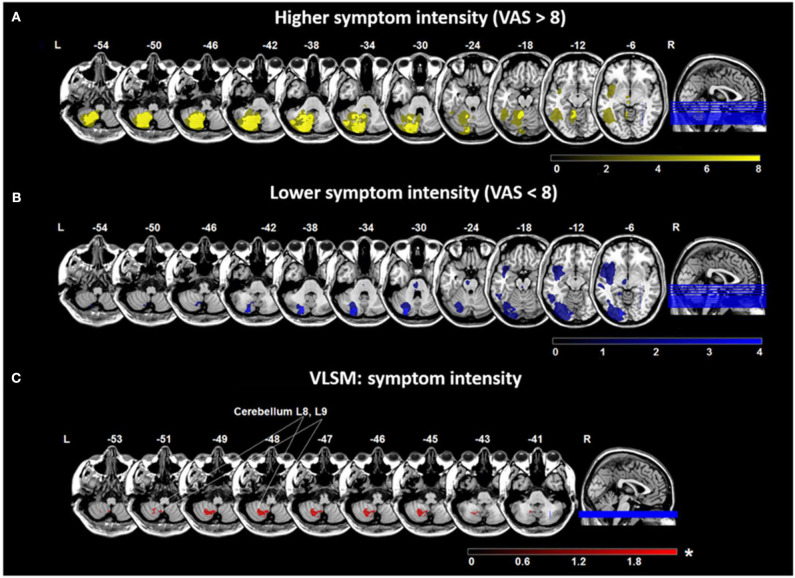
Overlap lesion plots and voxel-based lesion-symptom mapping (VLSM) for symptom intensity. Maximum intensity of the chief complaint was measured by the visual analog scale (VAS) (range 0–10). Overlap lesion plots for patients with higher symptom intensity (VAS > 8) (*n* = 34) **(A)** and lower symptom intensity (VAS < 8) (*n* = 13) **(B)**. The degree of lesion overlap is illustrated by color bars from dark to bright yellow or blue. **(C)** VLSM analysis in 47 patients based on VAS indicated an area in the medial cerebellar hemisphere (cerebellar layer 8, 9) related to more severe symptoms. Presented are voxels that exceed a *p* < 0.05 (*FDR-corrected). The color bar from dark to light red indicates the z-scores. Coordinates are given in MNI space. MNI, Montreal Neurological Institute; L, left; R, right.

The maximum symptom intensity (measured by VAS) was higher in the dizziness group (8.4 ± 1.8) and vertigo group (8.3 ± 2.3) and lower in the double vision group (6.2 ± 1.6) (*p* = 0.015 compared to the dizziness group and *p* = 0.04 compared to the vertigo group). Patients with nausea or vomiting had a higher VAS during the attack (9.5 ± 1.2), compared to patients without (7.0 ± 2.1) (*p* < 0.0001). VAS at symptom onset did not differ between patients with right-sided (7.7 ± 2.8) and left-sided lesions (8.2 ± 2.0) (*p* = 0.99). The mean decline of VAS per day was not significantly different between groups (dizziness group, 3.0 ± 1.7; vertigo group, 3.7 ± 1.7; double vision group, 1.9 ± 2.2). Lesions in patients with a high symptom intensity (VAS > 8) were larger and located in the cerebellar hemispheres (PICA > SCA territory) and pontomedullary brainstem (Figure 4A), while patients with a lower symptom intensity (VAS < 8) had smaller lesions in the cerebellar cortex (PICA/SCA territory), pontomesencephalic brainstem, thalamus and parieto-insular cortex (Figure 4B).

VLSM conducted with VAS at symptom onset showed significant voxels in the cerebellar layers 7b, 8, 9 in all patients with higher symptom intensity (*t-*test, *p* = 0.05 (FDR-corrected), Z = 1.75) (Figure 4C). In patients with cerebellar stroke, lesion volume was higher if symptoms were more severe (*r* = −0.42, *p* = 0.03), while in patients with cortical and thalamic lesions no correlation was found (*r* = −0.15, *p* = 0.85). Analysis of lesion volume and VAS at symptom onset by subgroups indicated no correlation for patients with vertigo (*r* = −0.1, *p* = 0.97), dizziness (*r* = −0.14, *p* = 0.63), or double vision (*r* = −0.14, *p* = 0.91).

### Lesion Topography and Symptom Duration

**Figure 5:**
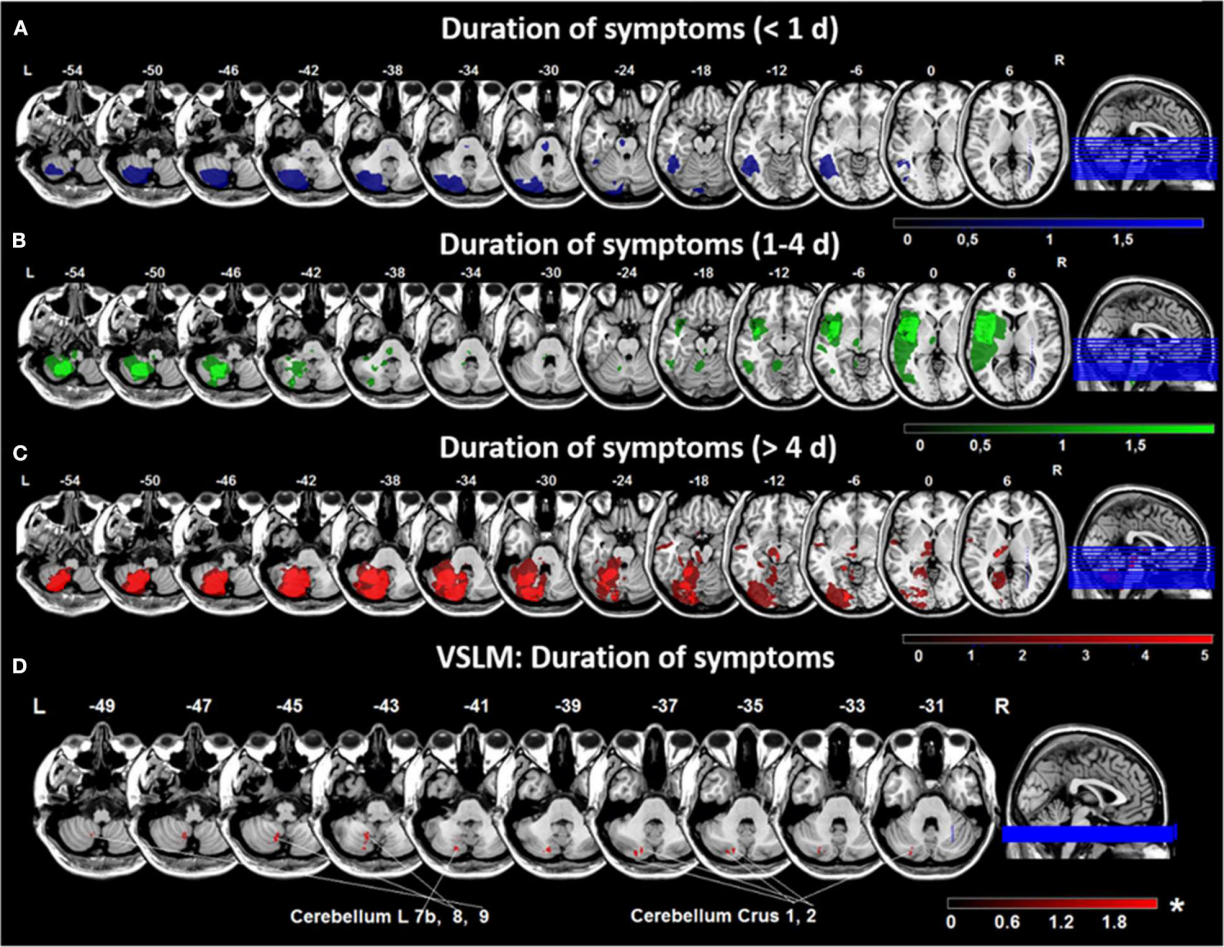
Overlap lesion plots and voxel-based lesion-symptom mapping (VLSM) for duration of symptoms. Overlap lesion plots are shown for patients with a duration of symptoms <1 day (*n* = 6) **(A)**, 1–4 days (*n* = 12) **(B)** and > 4 days (*n* = 29) **(C)**. The degree of lesion overlap is illustrated by color bars from dark to bright blue, green or red. **(D)** VLSM analysis in 47 patients based on symptom duration in days showed an area in the medial cerebellar hemisphere (cerebellar layer 7b, 8, 9, and Crus 1, 2) associated with longer duration of symptoms. Presented are voxels that exceed a *p* < 0.05 (*FDR-corrected). The color bar from dark to light red indicates the z-scores. Coordinates are given in MNI space. D, day; MNI, Montreal Neurological Institute; L. left; R, right.

In 6 patients symptom duration was <1 day, in 12 patients 1–4 days and in 29 patients >4 days. Duration of symptoms was not significantly different between the subgroups with vertigo, dizziness or double vision. In the total group, patients with a shorter symptom duration (<4 days) had lesions mostly in the lateral and distal cerebellar hemisphere (PICA territory), pontomesencephalic brainstem and parieto-insular cortex (Figures 5A,B), while patients with symptoms lasting >4 days had larger lesions involving the medial and lateral cerebellar hemispheres (PICA > SCA territory), the mesencephalon and thalamus (Figure 5C). Comparison of patients with a symptom duration of less and more than 4 days using VLSM showed that areas in the cerebellar layer 7b, 8, 9, and Crus 1, 2 were associated with longer symptom duration [*Liebermeister-*test, *p* = 0.05 (FDR-corrected), Z = 1.72] (Figure 5D).

### Lesion Distribution, QoL, and Functioning Parameters

The health-related QoL measured by the EQ-5D-5L questionnaire was worse in the vertigo (12.2 ± 4.0) compared to the dizziness group (9.4 ± 3.6; *p* = 0.02). In the vertigo group higher scores were found in the EQ-5D-5L subtests for mobility (*p* = 0.047), overall activity (*p* = 0.042), and anxiety (*p* = 0.024). Similarly, DHI was higher in the vertigo (52.0 ± 22.1) compared to the dizziness group (34.4 ± 20.2, *p* = 0.01) and lowest in patients with double vision (42.3 ± 27.9).

**Figure 6:**
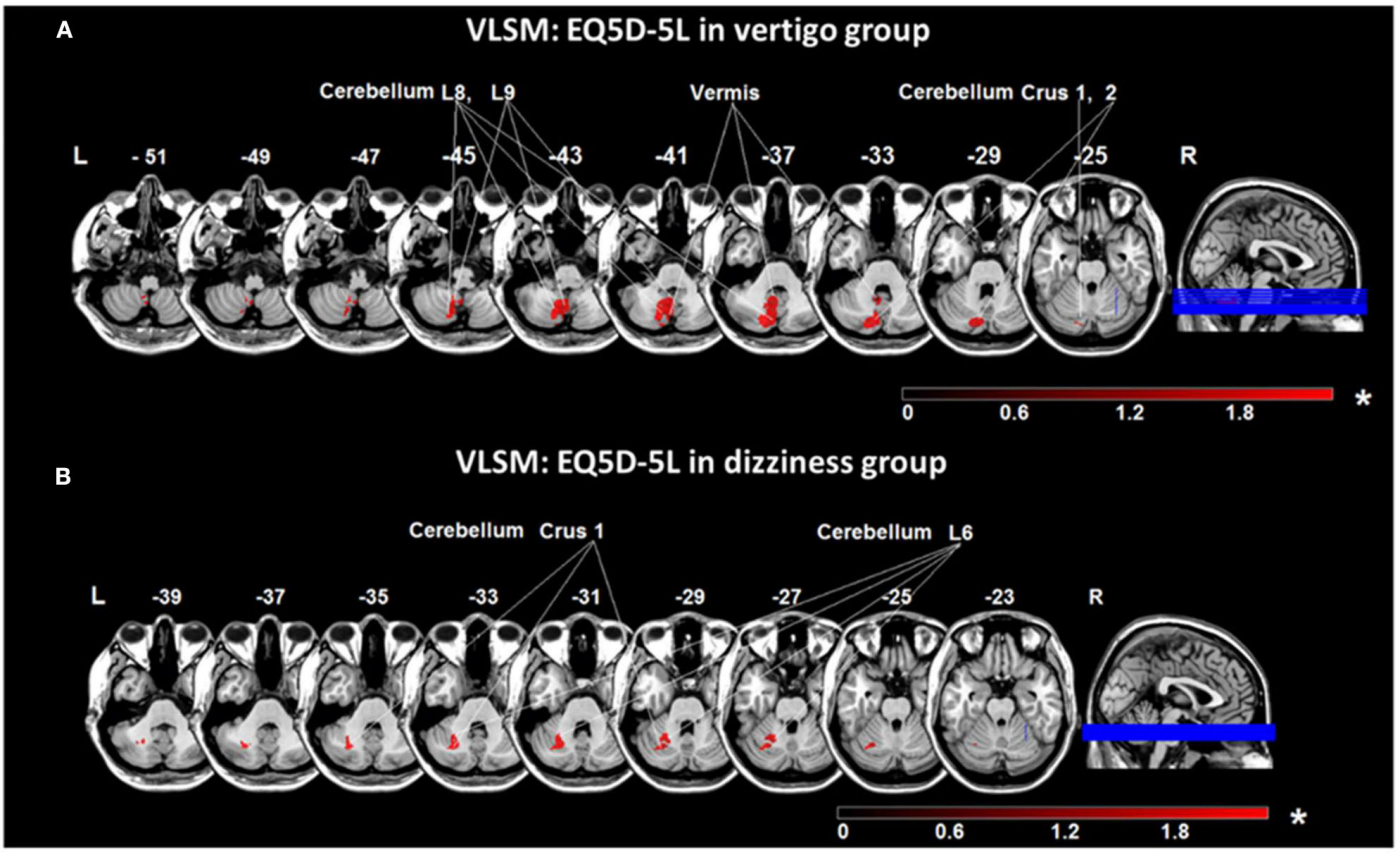
Voxel-based lesion-symptom mapping (VLSM) analyses based on the QoL scale EQ5D-5L. **(A)** VLSM based on EQ5D-5L in the vertigo subgroup showed an association of voxels in the medial cerebellar hemisphere (cerebellar layers 8, 9, vermis, and crus 1, 2) with worse QoL scores. **(B)** VLSM analysis using EQ5D-5L in the dizziness subgroup indicated a more lateral area (cerebellar layer 6, and Crus 1) engaged in higher EQ5D-5L scores. Presented are voxels that exceed a *p* < 0.05 (*FDR-corrected). The color bar from dark to light red indicates the z-scores. Coordinates are given in MNI space. EQ5D-5L: European Quality of Life scale −5 dimensions −5 levels, MNI, Montreal Neurological Institute; L, left; R, right.

VLSM in vestibular networks indicated that areas in cerebellar layers 8, 9, and Crus 2 were associated with higher EQ-5D-5L scores in the total group (*t*-test, *p* = 0.05 (FDR-corrected), Z = 1.69). VLSM for the subtest anxiety/depression showed a significant specific engagement of cerebellar layer 8 [*t*-test, *p* = 0.05 (FDR-corrected), Z = 1.79]. The analysis within patient subgroups revealed that the cerebellar layer 6 and Crus 1 correlated with worse QoL in patients with vertigo (Figure 6A), while the cerebellar layer 8 was related to higher EQ-5D-5L scores in patients reporting dizziness (Figure 6B). When VLSM was performed for DHI, areas in cerebellar layer 8 were significantly associated with higher DHI scores.

## Discussion

In the prospective EMVERT lesion trial, symptoms of patients with acute vestibular or ocular motor stroke were systematically documented and correlated to lesion topography. The major findings were the following: (1) Vertigo and dizziness were equally frequent in vestibular stroke and underlying lesions showed a large overlap in the cerebellar hemisphere. Vertigo was more likely associated with medial cerebellar lesions (biventer lobule, nodulus, uvula), while dizziness appeared more frequently in lateral and superior cerebellar lesions. (2) Symptom intensity and duration varied largely in vestibular and ocular motor stroke patients. Higher symptom intensity and longer symptom duration were associated with medial cerebellar lesions. Cortical lesions presented with milder symptoms of shorter duration. (3) QoL and functioning was worst in patients with vertigo and lesions in the medial cerebellar structures.

### Symptom Characteristics and Diagnostic Classification of Vestibular Stroke

The diffuse lesion-symptom topography in vestibular and ocular motor stroke has direct practical implications for the processing of patients. The symptom quality does not allow differentiation of peripheral and central etiologies of vestibular syndromes. Therefore, the traditional approach of assessment by the symptoms vertigo, dizziness, postural instability or disequilibrium has major limitations in acute vestibular disorders (16, 17). Similar conclusions have been drawn in previous studies, where symptom quality was imprecise even in peripheral vestibular disorders (18). In the current study, symptom duration and intensity varied largely and consequently could not be taken as an indicator or criterion for exclusion of stroke. A recent study showed that functional impairment in acute central vestibulopathies is lower than in acute unilateral peripheral vestibulopathies, which may increase the risk of a false-benign diagnosis in vestibular stroke (19). Furthermore, previous studies described that suspected ischemic attacks with vestibular symptoms may present with short-lasting and transient symptoms (50% lasting <1 h) (20–22). Consequently, modern concepts of symptom-based differentiation of vestibular disorders rely more on the presence of triggers preceding vestibular symptoms and the time course of symptom onset and evolution (e.g., TiTrATE algorithm, including timing, trigger, and targeted examination) (23, 24).

### Symptom Characteristics and the Risk of Misdiagnosis of Vestibular Stroke

Based on a recent meta-analysis, unspecific presentations of dizziness, short duration, and subtle intensity of symptoms may increase the risk for a misdiagnosis in patients with acute vestibular stroke (5). In these scenarios, the probability for missing stroke was about 10-fold compared to other focal neurological presentations. In total about 10% of strokes were missed at first contact in the ED (25). This problem is also reflected in a 50-fold increased risk of being readmitted to a hospital with a secondary stroke diagnosis in the first week, and a 9.3-times higher stroke risk after 30 days in patients discharged from the ED with a suspected benign diagnosis of acute vertigo or dizziness compared to matched controls (26). Lesion-symptom relationships from the current EMVERT lesion trial point out that especially patients with lesions in the lateral cerebellar hemispheres, mesencephalon and parieto-insular cortex may be at risk of being falsely processed. In these localizations patients do complain about more unspecific symptoms (such as dizziness, unsteadiness), transient symptoms (<1 day), lower symptom intensity (VAS < 8), and less vegetative symptoms (like nausea or vomiting) (Figures 2, 3, 5). Furthermore, patients with lesions in the lateral cerebellum, upper midbrain and cortex do not show clinical signs of an acute vestibular syndrome (e.g., SPN), which further complicates the diagnosis. HINTS is not applicable in the majority of these cases. In contrast, lesions in the pontomedullary tegmentum and medial cerebellar hemispheres may be more apparent, because patients report more intense and longer-lasting symptoms and show more prominent clinical signs (such as SPN, ocular tilt reaction, and HINTS central pattern) (27–29). Lesion size may be another relevant factor, because patients with smaller lesions had less intense vertigo. For lesions <10 mm, MRI has a high false-positive rate (about 50%) in the first 1–2 days after symptom onset, which questions the rationale of a purely imaging-based diagnosis of acute vestibular or ocular motor stroke (30). Patients in our study received MRI in 93% of cases later than 1 day post symptom onset to increase the sensitivity to capture small DWI lesions.

### Pathophysiological Principles Behind Lesion-Symptom Relationships in Vestibular Stroke

Despite the variety of symptomatic presentations across lesion sites, some general principles seem to exist: (1) Lesions in the nodulus, uvula, and medial cerebellar hemisphere are associated with vertigo symptoms of the highest intensity and a high rate of nausea or vomiting. The most likely explanation is that these regions are directly involved in processing of vestibular and ocular motor signals. The nodulus has been implicated in integration of otolith and semicircular canals signals, tilt suppression of post-rotatory vertigo and the judgement of verticality perception (7, 31–33). Nodular lesions often present with SPN and ocular tilt reaction (34). Anatomically, the nodulus has inhibitory ipsilateral projections to the vestibular nucleus (31). Functionally, medial cerebellar lesions cause an excitation of the ipsilesional vestibular nucleus (via disinhibition) and resemble the clinical picture of a vestibular nucleus lesion on the other side. The lateral and superior cerebellar hemispheres are not specifically dedicated to vestibular processing but rather to sensorimotor and posture control. Therefore, lesions may cause less specific dizziness, as a sign of disturbed multisensory integration or balance control, and only rarely nausea or vomiting. (2) Perceived impairment of QoL and functioning follows the degree of vestibular asymmetry. Patients with vertigo had a higher EQ-5D-5L anxiety score than dizzy patients. VLSM found an association of higher EQ-5D-5L scores in the medial cerebellar hemisphere. In accordance, a recent study found that the degree of horizontal SPN is the most important factor for worse health-related quality of life in acute vestibulopathies (19). In another previous study, patients with unilateral vestibular disorders had more anxiety than patients with bilateral vestibulopathy (35). (3) Symptom duration was higher in medial compared to lateral cerebellar and thalamo-cortical lesions. This finding could be explained either by the different peak levels of initial symptoms in these subgroups or by a less effective central compensation of strategic lesions in vestibular cerebellar networks. The latter hypothesis may be substantiated by the finding that patients with medial cerebellar lesions had a prolonged course of compensation (36). Furthermore, symptoms from unilateral parieto-insular cortex lesions may be compensated by the intact cerebral hemisphere (6). (4) Symptom quality changed along the brainstem-thalamic axis from more direction-specific symptoms (i.e., vertigo) in the lower brainstem to more position-specific symptoms (i.e., dizziness) in the midbrain and thalamus. The reason for this topography may be the specific computation of vestibular signs at different brain levels. Vestibular signs at the lower brainstem level drive direction-specific ocular motor and postural responses. Along the ascending vestibular projections head direction signals from both sides are integrated to head position in space signals (37, 38). In the thalamo-cortical networks, a global percept of the environment is built by integration of multisensory information. In consequence, lesions at the midbrain level and above will rather give dizziness as a disturbed perception of the environment without the feeling of self-motion (6).

## Conclusions

A simple symptom-lesion topography in acute vestibular and ocular motor stroke is an inappropriate clinical approach. Symptom quality, intensity, and duration are not suited to differentiate peripheral from central etiologies of vestibular presentations. Clinicians should be aware that rare lesion sites in the lateral cerebellum, thalamus, or cortex may present with rather unspecific, mild, and transient symptoms and therefore are at risk of being categorized as false-benign. Symptom intensity and perceived impairment are highest in lesions, which directly affect pontomedullary and medial cerebellar vestibular hubs. Lesions in ascending vestibular projections above the VOR brainstem circuit, rarely present with direction-specific vestibular symptoms (namely vertigo). Detailed neuro-ophthalmological and -ototological examinations are required in all patients with monosymptomatic vertigo, dizziness, or double vision.

## Data Availability Statement

The raw data supporting the conclusions of this article will be made available by the authors, without undue reservation.

## Ethics Statement

The studies involving human participants were reviewed and approved by Ethics Committee of the University of Munich on 02/23/2015 (57-15). The patients/participants provided their written informed consent to participate in this study.

## Author Contributions

AZ: drafting/revising the manuscript, study concept and design, acquisition of data, analysis and interpretation of data, and statistical analysis. KM: drafting/revising the manuscript, study concept and design, acquisition of data, and analysis and interpretation of data. ES: drafting/revising the manuscript, analysis and interpretation of data, and statistical analysis. HH: drafting/revising the manuscript and acquisition of data. TB and MD: revising the manuscript, study concept and design, and analysis and interpretation of data. KJ: drafting/revising the manuscript, study concept and design, and analysis and interpretation of data. All authors contributed to the article and approved the submitted version.

## Conflict of Interest

The authors declare that the research was conducted in the absence of any commercial or financial relationships that could be construed as a potential conflict of interest.

## Abbreviations

AICA: Anterior Inferior Cerebellar Artery
BPPV: Benign Peripheral Positional Vertigo
CT: Computed Tomography
DHI: Dizziness Handicap Inventory
DWI: Diffusion-Weighted Image
EMVERT: EMergency VERTigo and balance disorders
EQ-5D-5L: European Quality of life scale–5 Dimensions–5 Levels
EQ-VAS: European Quality of life scale—Visual Analog Scale
FLAIR: Fluid Attenuated Inversion Recovery
FSPGR: Fast Spoiled Gradient Echo
INO: internuclear ophthalmoplegia
MNI: Montreal Neurological Institute
MRI: Magnetic Resonance Imaging
mRS: modified Rankin Scale
PICA: posterior inferior cerebellar artery
SCA: superior cerebellar artery
T: Tesla
VAS: Visual Analog Scale
VLSM: Voxel-based Lesion-Symptom Mapping
VOR: Vestibulo-Ocular Reflex.
