# Supplementary material for: A Prospective Analysis of Lesion-Symptom Relationships in Acute Vestibular and Ocular Motor Stroke
Source: Front Neurol. 2020 Aug 6;11:822. doi: 10.3389/fneur.2020.00822 (PMC7424024; doi:10.3389/fneur.2020.00822)
Supplement: Supplementary file 1 [file Image_1.pdf]

SUPPLEMENTAL MATERIAL

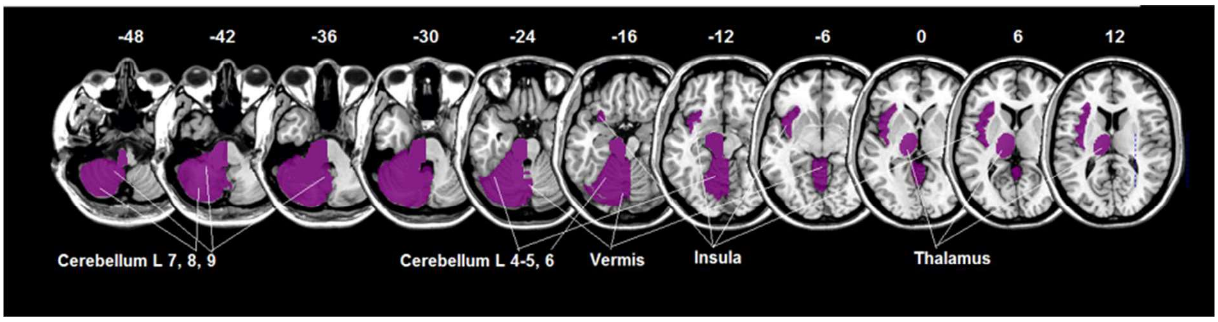

Online Supplement 1: **Brain areas included in the custom-made mask.** The mask includes all relevant hubs of the central vestibular and ocular motor networks.
