# Supplementary material for: A Prospective Analysis of Lesion-Symptom Relationships in Acute Vestibular and Ocular Motor Stroke
Source: Front Neurol. 2020 Aug 6;11:822. doi: 10.3389/fneur.2020.00822 (PMC7424024; doi:10.3389/fneur.2020.00822)
Supplement: Supplementary file 2 [file Table_1.pdf]

		Vertigo	Dizziness	Double Vision
PICA	**PICA territory total**	**58%**	**35%**	**12%**
	Nodulus	37%	-	-
	Uvula	32%	5%	-
	Pyramis	5%	-	-
	Flocculus	10%	-	-
	Tonsil	32%	-	-
	Biventer lobule	58%	25%	-
	Inferior semilunar lobule	37%	15%	12%
	Superior semilunar lobule	5%	25%	-
SCA	**SCA territory total**	**10%**	**15%**	-
	Central lobule	10%	5%	
	Lingula	10%	5%	
	Post. quadrangulate lobule	5%	15%	
	Ant. quadrangulate lobule	10%	15%	
Brainstem	**Brainstem total**	**37%**	**40%**	**88%**
	Medulla	16%	-	
	Pons	21%	20%	25%
	Midbrain	5%	25%	75%
Thalamus	**Thalamus total**	**5%**	**10%**	**25%***
	Dorsolateral thalamus	5%	5%	-
	Anteromedial thalamus	-	5%	25%*
Cortex	**Cortex total**	**10%**	**15%****	
	Parieto-insular cortex	10%	-	-
	Occipital cortex	-	15%**	-

Online Supplement 2: Involvement of vascular territories and anatomical structures in the different chief complaints in vestibular and ocular motor stroke. For the chief complaint vertigo, the most frequent lesion sites were in the medial PICA territory (nodulus, uvula, tonsil, medial biventer, inferior semilunar lobules) and pontomedullary brainstem, for dizziness in the lateral PICA territory (lateral biventer, inferior semilunar, superior semilunar lobules), SCA territory (posterior/anterior quadrangulte lobules), and the pontomesencephalic brainstem and for double vision in the mesencephalic brainstem/thalamus. Data are shown as % of all patients with the respective chief complaint. *All patients with double vision and anteriomedial thalamic lesions also had midbrain lesions. **All patients with lesions in the occipital cortex had also lesions in the brainstem or cerebellum.
